# Supplementary material for: Optimization of power management in PV-based smart grids using grid-support and grid-forming inverters
Source: PLoS One. 2026 Feb 12;21(2):e0338191. doi: 10.1371/journal.pone.0338191 (PMC12900292; doi:10.1371/journal.pone.0338191)
Supplement: Attachment — Submitted filename: Postdoc-plos one Reviewer Comments.docx [file pone.0338191.s002.docx]

**Response to Reviewer #1**

We sincerely thank the reviewer for the constructive comments and insightful suggestions that have helped us improve the quality and clarity of our manuscript. We have carefully revised the paper in light of the provided feedback. Below, we provide a point-by-point response.

1. **Literature Review (Outdated References)**

Response: We agree with the reviewer that including more recent studies will enhance the relevance of our work. Accordingly, we have updated the literature review by incorporating recent works from 2023–2025, with a particular focus on **Virtual Synchronous Generators (VSGs), AI-driven control strategies, and distributed storage optimization.** These updates provide a more comprehensive context for our research contributions.

2. **Introduction and PV Output Modeling Approaches**

Response: We have expanded the introduction to include a discussion of **uncertainty modeling approaches** for PV output, including stochastic programming, scenario-based techniques, and Conditional Value-at-Risk . We also referenced the suggested studies (A Distributed Market-Aided Restoration Approach of Multi-Energy Distribution Systems Considering Comprehensive Uncertainties from Typhoon Disaster and Risk-averse stochastic capacity planning and P2P trading collaborative optimization for multi-energy microgrids considering carbon emission limitations: An asymmetric Nash bargaining approach` ) to strengthen the background and clarify the novelty of our approach compared to mainstream methodologies.

3. **Optimization Objective Function (Broader Scope)**

Response: We acknowledge the reviewer’s concern and have revised the optimization objective function discussion to include considerations of **frequency stability, battery lifetime, and economic trade-offs.** Additionally, we have added a **sensitivity analysis in Section 7** to illustrate the impact of these factors on system performance. This provides a more holistic view of the optimization strategy.

4. **Simulations and Stress Tests**

Response: We appreciate this valuable suggestion. We have extended the simulations to include more realistic stress scenarios, such as **prolonged irradiance drops and significant load variations**, to demonstrate better the robustness of the proposed control strategies under challenging operating conditions.

5. **Parameter Justification (Table 1)**

Response: We agree that parameter justification is necessary for transparency. We have revised the manuscript to clarify that the parameter values in Table 1 are derived from a combination of **industry-standard specifications, established literature, and reasonable assumptions for research simulations.**

6. **Figure Readability and Explanations**

Response: Thank you for pointing out the clarity issues. We have revised Figures 9–12 to **improve labelling, separate overlapping curves, and enhance legends** for better readability. Additionally, we have expanded the figure captions and added detailed explanations in the text to highlight the key phenomena being illustrated.

7. **Limitations Section (Expanded)**

Response: We acknowledge that the original limitations section was insufficiently comprehensive. We have now expanded it to include discussions on **communication delays, coordination challenges in multi-inverter systems, and practical issues related to scaling up to large smart grids**. These additions strengthen the realism and balance of the study.

**Response to Reviewer #2**

We sincerely thank the reviewer for the constructive comments and valuable suggestions that have helped us identify key areas for improvement in our manuscript. We have carefully revised the paper and addressed each point as follows:

1. **Introduction (Defects of Existing Research and Comparative Analysis)**

*Response:* We acknowledge the reviewer’s concern and have expanded the introduction to clearly elaborate on the shortcomings of existing research under high PV penetration, such as challenges in stability, coordination, and optimization. We now include a comparative analysis showing how the proposed strategies—particularly the combination of grid-support and grid-forming inverters with real-time power management—overcome these gaps. This addition strengthens the necessity and positioning of our research.

2. **System Architecture and EMS Logic**

*Response:* We have revised Section 3.3 to provide a more detailed description of the Energy Management System (EMS), including its control logic for real-time coordination of PV generation, storage, and inverters. Flowcharts and additional explanations have been added to clarify how the EMS sets parameters dynamically and responds to fluctuations. The basis for key parameter selection has also been explained with supporting references.

3. **Grid-Support Inverter Configuration (Equation 2 and Terminology)**

*Response:* We thank the reviewer for pointing out the ambiguity. Equation (2) has been revised for clarity, and the term “demerit reactive power” has been corrected to “compensated reactive power.” We also clarified the correlation mechanism between reactive power and voltage regulation, showing how reactive power injection/absorption influences voltage stability.

4. **Equation (4) Correction (Grid-Forming Inverter)**

*Response:* We apologize for the error. Equation (4) has been corrected to properly define active power as the product of voltage, current, and power factor. The revised equation now ensures accuracy in subsequent analyses, and the affected parts of Section 3.2 have been updated accordingly.

5. **Objective Function (Equation 6) Definitions**

*Response:* We agree that the objective function lacked clarity. The mathematical definitions and parameter descriptions of all cost terms ( *Closs*, *Cstorage*, *Cvoltage*) have now been explicitly included.

6. **Simulation Environment (Model Details and Experimental Validation)**

*Response:* We have revised Section 6.1. to include detailed descriptions of the MATLAB/Simulink models used for PV modules, storage systems, and inverters, with appropriate references. While full-scale hardware testing is beyond the current scope, we have now included comparisons with benchmark results from existing experimental studies to increase credibility. Additionally, we discuss in the limitations that future work will involve hardware-in-the-loop (HIL) validation.

7. **Case Study 1 (Figure 9 and Data Sources)**
   *Response:* We have corrected Figure 9 by adding power units (kW).
8. **Discussion Section (Extreme Conditions and Delay Impacts)**

*Response:* We have expanded the discussion to analyze the adaptability of the strategy under extreme weather conditions, including prolonged rainy/cloudy periods. We also included a discussion of the impact of inverter coordination delays on system stability and how robust control strategies and improved communication protocols may mitigate these effects.

9. **Limitations (Cost and Capacity Optimization of Storage)**

*Response:* We appreciate this suggestion. The limitations section has been expanded to provide specific cost ranges (based on current Li-ion battery technology) and capacity sizing methodologies derived from optimization studies. This provides clearer guidance for future research directions in cost-effective and scalable energy storage integration.

In summary, we have addressed the reviewer’s concerns by revising the introduction, correcting mathematical expressions, clarifying system logic, detailing model assumptions, improving simulation scenario explanations, and expanding the discussion and limitations with more depth and specificity. These revisions substantially improve the rigour, clarity, and novelty of the manuscript.

Sincerely,

**Corresponding Author**

**Dr. Amam Hossain Bagdadee**
